# Comprehensive Analysis of *Betula platyphylla* Suk. PIF Gene Family and Their Potential Functions in Growth and Development

**DOI:** 10.3390/ijms232315326

**Published:** 2022-12-05

**Authors:** Aihua Chen, Peng Huang, Shanshan Guo, Sige Liu, Xiaoqing Hu, Xuemei Liu

**Affiliations:** 1 College of Life Science, Northeast Forestry University, Harbin 150040, China; 2 Key Laboratory of Non-Wood Forest Product Research and Development, Mudanjiang Branch of Heilongjiang Academy of Forestry, 16 Diming Street, Mudanjiang 157011, China; 3 Key Laboratory of Saline-Alkali Vegetation Ecology Restoration, Ministry of Education, Northeast Forestry University, Harbin 150040, China

**Keywords:** *Betula platyphylla*, phytochrome-interacting factors, PIF, basic helix–loop–helix (bHLH), adventitious root

## Abstract

Phytochrome-interacting factors (PIFs) are transcription factors with the basic helix–loop–helix (bHLH) domain. As integration factors between different signal pathways, members of the PIF protein family regulate many aspects of plant growth and development, such as seed germination, photomorphogenesis, thermomorphogenesis, rhythm regulation, flowering response, stomatal development, and stress responses. Our previous studies have shown that the *BpSPL2* gene may regulate plants’ adventitious root development through *PIF* genes. Within the *Betula platyphylla* genome, we identified eight *PIF* (*BpPIFs*) genes. We analysed and named them based on a phylogenetic tree, gene structures, and conserved motifs. Synteny analysis indicated that transposition or segmental duplication events played a minor role in the expansion of *BpPIFs*. The comparative syntenic analysis combined with phylogenetic analysis provided a deep insight into the phylogenetic relationships of *BpPIF* genes, suggesting that *BpPIF* proteins are closer to *PtPIF* than to *AtPIF*. The analysis of cis-acting elements in promoter regions of *BpPIF* genes indicated that various elements were related to light, abiotic stress, and plant hormone responsiveness. In addition, we found that these promoters have the transcription factor of *B. platyphylla SPL2* (*BpSPL2*) binding motif GTAC. Expression analysis demonstrated that *BpPIF* genes, especially *BpPIF4*, *BpPIF9b*, and *BpPIF10,* might be the potential target genes of *BpSPL2* in the process of adventitious root formation. Besides providing a comprehensive understanding of the *BpPIF* family, we propose a hypothetical gene network regulatory model for adventitious root formation.

## 1. Introduction

Transcription factors (TFs) play a crucial role in responses to environmental cues through self-regulation and the regulation of downstream target gene expression, forming a complex network of signal transduction pathways [1]. For example, light is an essential environmental signal in plant growth and development. Plants can perceive the intensity, length, and direction of the light through the photoreceptors in their structures, including cryptochrome (Cry) and phototropin (Phot) for sensing blue light, phytochrome (PhyA-E) for sensing red and far-red light, and UV for sensing ultraviolet-blue light-B receptor (UV-B receptor) [2,3]. Plant phytochromes can occur either in the biologically active far-red light absorbing form (Pfr) or in the non-biologically active red-absorbing form (Pr). These two photosensitive pigments can be converted into each other after absorbing red and far-red light, triggering plants’ morphogenesis [4].

Like phytochromes, their primary signalling partners (PHYTOCHROME-INTERACTING FACTORs-PIFs) have been discovered from bryophytes to angiosperms. PIFs, which belong to the 15th subfamily of the bHLH gene family, are one of the downstream elements co-regulated by photoreceptors that directly interact with phytochrome B (phyB) [5,6]. In addition, PIFs have been shown to mediate metabolic signals to the circadian clock, thermomorphogenesis, hormone signalling, biotic and abiotic responses.

In *Arabidopsis thaliana*, eight *PIFs* were found and named *PIF1*-*PIF8*. All these *PIFs* have an APB motif, while only *PIF1* and *PIF3* have both an APB and an APA motif. *PIF1*/*PIF3*-*LIKE* 5 (*PIL5*), *PIF3*, *PIF4*, *PIF5*/*PIL6*, *PIF6*/*PIL2* and *PIF7* combine with phyB through the N-terminal conserved domain to form a complex [7,8,9]. After binding to phyB, *PIFs* are rapidly phosphorylated and then degraded by the proteasome through ubiquitination. *PIFs* have been found to bind sequence-specifically to a DNA G-box core motif (CACGTG). Moreover, *PIFs* can form homodimers or heterodimers to enhance DNA binding, suggesting a direct signalling pathway to regulate the expression of their target genes by enhancing or inhibiting the DNA-binding activity [9].

*PIFs* function primarily as negative regulators of photomorphogenesis. In Arabidopsis, *PIF1* is known to repress seed germination in darkness [10,11]. PhyA/PhyB is deactivated under far-red light, causing *PIF1* to accumulate and thus induce SOMNUS (SOM) expression, which promotes the expression of the MOTHER OF FT AND TFL1 (MFT) gene, a germination inhibitor. Then, abscisic acid (ABA) and gibberellin (GA) accumulation reach a dynamic balance, leading to the repression of seed germination through a mechanism involving ABI5 and DELLA proteins [12,13,14,15,16]. *CTG10* interacts with *PIF1*, forming a feedback loop of *CTG10*/*PIF1*, which can also participate in light-dependent seed germination [17].

*PIF3*-*LIKE1* (PIL1), renamed *PIF2* later, interacts with *HFR1* and *PIFs* (*PIF1*, *PIF3*, *PIF4*, and *PIF5*) and coordinates with *HFR1* to suppress the transcriptional activity of *PIFs*, thus promoting photomorphogenesis [18]. *PIF3* was the first identified gene in the *PIF* family by a yeast two-hybrid screen for phyB-interacting proteins [19]. *PIF3* participate in light-responsive transcriptional network genes in coordination with the plant hormones and the circadian clock, modulating plant growth and development [20,21]. *PIF3* can also enhance the freezing tolerance of plants by a regulatory module CBFs-PIF3-phyB [22].

*PIF4* acts as a hub integrating light and temperature cues, inducing endogenous hormonal signalling and driving skotomorphogenesis, photomorphogenesis and thermomorphogenesis [23]. Arabidopsis controls shade avoidance syndrome (SAS) to mediate the hypocotyl elongation by PIF4-SHY2 modules [24]. *PIF4* participates in dark-triggered and brassinosteroid-induced leaf senescence [25]. ZTL induces the expression of *YUC8* in upper aerial parts and promotes hypocotyl elongation via *PIF4* [26]. *PIF5* often cooperates with *PIF4* to perform its functions, such as regulating axillary branching via stem auxin signalling and bud abscisic acid [27]. Anthocyanin accumulation is a striking symptom of plant environmental response and plays a key role in plant adaptation to adverse stimuli [28]. Elevated temperature activates *PIF4* to stimulate auxin signalling, which causes hypocotyl elongation and leaf hyponasty [29]. *COR27* up-regulates its expression in a circadian clock-dependent manner and controls hypocotyl elongation [30]. In Arabidopsis seedlings, *PIF4* positively regulates the development of stomata and negatively regulates anthocyanin accumulation [31]. *PIF4, PIF5*, and *PIF7* regulate shade avoidance, affecting the elongation of hypocotyls by controlling photoperiod [32,33].

*PIF6* produces two splice variants, α and β, of which the β-form regulates seed dormancy. As it is ectopically overexpressed and under continuous red light, PIF6 inhibits hypocotyl elongation [34]. The function of *PIF7* is similar to that of *PIF4*, acting in the shade response [35,36,37,38,39]. *PIF7* and PIF3, together with PIF4, function additively to promote hypocotyl elongation under continuous red light by suppressing phyB levels [40]. Under shade conditions, *PIF7* also regulates shade avoidance responses by directly controlling auxin biosynthetic genes [41]. Like *PIF4* [42], *PIF7* is also involved in the flowering of *Arabidopsis thaliana* [43]. In rose flowers, the PIF8-BBX28 module can regulate petal senescence by governing mitochondrial ROS homeostasis at night [44]. In addition, the Populus homolog PIF8 plays a major role as a suppressor of seasonal growth [45].

Recently, studies have revealed that bHLH transcription factors (PFA and PFB proteins) participate in the formation of lateral root primordia [46] and that the bHLH transcription factor *PIF4* controls the flowering time by activating *FT* under increasing temperature [44].

The *SPL* gene family and the *PIF* gene family interact with each other to regulate the growth and development of plants. In Arabidopsis, we found that PIF4 interacts with SPL9 to inhibit shoot branching [47]. The transcription factors PIF/PIL interact with SPLs and play a conserved role in repressing tillering/branching in wheat, rice, and Arabidopsis [47]. However, the functional significance of the phytochrome–PIF relationship is not fully understood in birch, especially in the process of adventitious root development. Therefore, in this study, we identified eight *BpPIF* genes in *B. platyphylla* and analysed them comprehensively, including the gene structure and motif compositions, synteny analysis and gene duplications, phylogenetic relationship, conserved promoter motifs and candidate transcription factors which might directly bind the promoter of *BpPIFs*, which were further investigated. In addition, based on RNA-seq data, the *BpPIF* gene expression profiles in the adventitious root occurrence of *B. platyphylla* transgenic *BpSPL2* lines and male flower development were determined. Furthermore, the expression levels of *PIF* gene family members during the development of adventitious roots were studied by RT-PCR. Finally, we obtained candidate target *BpPIF* genes of *BpSPL2* during the adventitious root formation of *B. platyphylla* and developed a hypothetical network regulation model.

## 2. Results

### 2.1. Genome-Wide Identification and Analysis of PIF Genes in Betula platyphylla Suk

To identify PIF family genes in the *B. platyphylla* genome, we downloaded the Arabidopsis, Populus and Betula genome data from the Phytozome database [48]. We employed eight *Arabidopsis thaliana* PIF proteins (Table S1), ten putative *Populus trichocarpa* PIF proteins [49] (Table S2) and the consensus protein sequences of bHLH (PF00010: TEVHNRSERKRRDRINEKMKALQELIPHCNKTDKASMLDEAIEYMKSLQL) as a query to search against the *B. platyphylla* genome databases using the BlastP program. After removing redundant proteins, 31 candidate proteins were obtained (Table S3). To confirm the presence of the bHLH domain in those putative *B. platyphylla* PIF proteins (BpPIFs), their amino acid sequences were searched using Pfam and Web CD-search Tool (Table S4). Based on the Betula database from Phytozome, we infer that the *B. platyphylla* PIF gene family contains seven members: BPChr06G16498, BPChr05G27408, BPChr11G17797, BPChr04G09407, BPChr12G25898, BPChr08G16198, and BPChr13G16040. Nevertheless, in our transcriptome sequencing data, we identified eight BpPIF family members (Table 1): Bpev01.c0015.g0022.mRNA1, Bpev01.c1527.g0004.mRNA2, Bpev01.c1708.g0006.mRNA1, Bpev01.c0349.g0043.mRNA1, Bpev01.c0555.g0004.mRNA1, Bpev01.c0918.g0013.mRNA1, Bpev01.c1013.g0001.mRNA1, and Bpev01.c0000.g0060.mRNA1. We then analysed these members’ molecular weights (MWs) and isoelectric points. The MWs of these BpPIF proteins ranged from 39.40 kDa (BpPIF9a) to 79.48 kDa (BpPIF3), and their PIs ranged from 5.01 (BpPIF9a) to 9.25 (BpPIF7).

### 2.2. Phylogenetic Analysis of the BpPIF Gene Family

**Figure 1:**
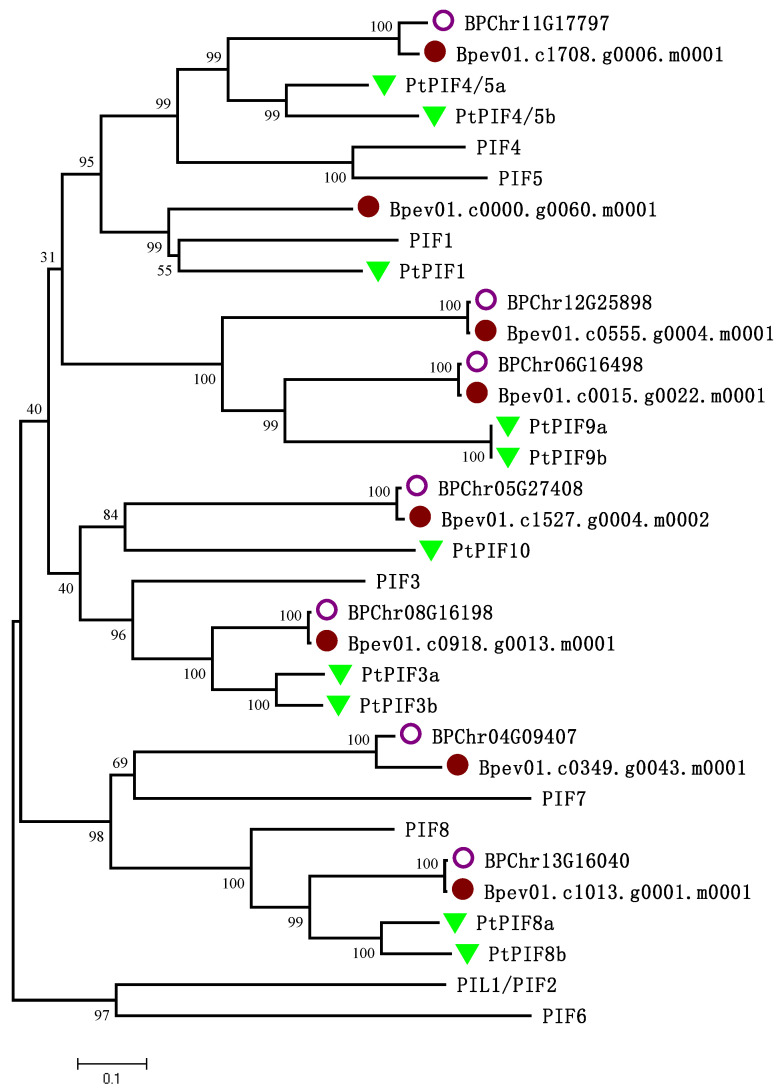
The evolutionary history was inferred using the neighbour-joining method. Then, the phylogenetic tree was constructed based on the full-length protein sequences of BpPIFs, 8 AtPIF, and 10 PtPIF proteins using MEGA 7.0 software (available online: https://www.megasoftware.net/, accessed on 29 August 2021).

In order to better understand the evolutionary relationship between Betula (*B. platyphylla* and *B. pendula*)*,* Arabidopsis, and Poplar, the evolutionary tree was inferred using the neighbour-joining method using bootstrap analysis (1000 replicates) from alignments of the PIF complete protein sequences from *BpPIFs*, 8 *AtPIFs* and 10 *PtPIFs* (Figure 1, Table 1**)**. Based on these evolutionary relationships, we renamed these genes as *BpPIF1, BpPIF3, BpPIF4, BpPIF7, BpPIF8, BpPIF9a, BpPIF9b,* and *BpPIF10.* Compared with the members of the three species *PIF* gene family, *BpPIF1, BpPIF3, BpPIF4, BpPIF7, and BpPIF8,* they are evolutionarily conserved and closely related. Interestingly, in poplar and birch, a common branch different from Arabidopsis appeared, which we named *BpPIF9a, BpPIF9b, and BpPIF10.* Thus, the *BpPIF* family of birch is more closely related to the *PtPIF* family of poplar.

### 2.3. Gene Structure and Conserved Motif Analysis of BpPIF Gene Family

**Figure 2:**
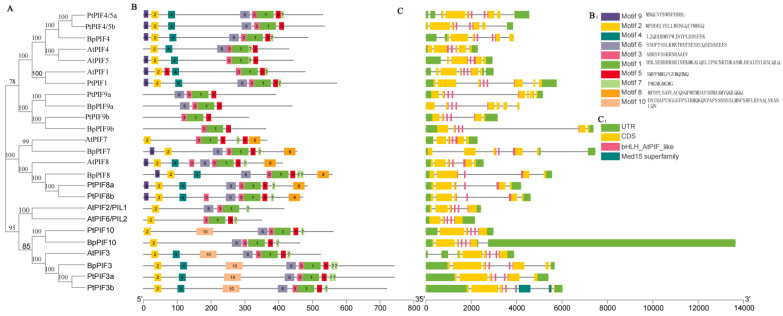
Phylogenetic relationships, conserved protein motifs, and gene structure in *PIF* genes from *Betula platyphylla* Suk. (**A**) The phylogenetic tree of *Betula platyphylla* Suk., *Arabidopsis thaliana,* and *Populus trichocarpa* PIFs were exhibited. (**B**) The motif composition of BpPIF proteins. The motifs are displayed in different coloured boxes. B_1_ is the legend of Figure B (**C**) The exon–intron structure of *BpPIF* genes. Exons and UTRs are represented as dark yellow and green boxes, respectively, while black lines indicate introns. Pink boxes highlight the bHLH-AtPIF-like domain. C_1_ is the legend of figure C.

To support the phylogenetic analysis, we performed a gene structure analysis of *BpPIF* family members from *Betula platyphylla*, *Arabidopsis thaliana,* and *Populus trichocarpa*. As shown in Figure 2C, the numbers of exons in *BpPIF*, *AtPIF* and *PtPIF* genes were conserved, ranging from five to eight exons. The number of exons of the birch *PIF* genes was precisely the same as the corresponding poplar *PIF* genes. We found that the gene structures of putative *BpPIF* members were highly conserved in all three species. The number of introns contained in their bHLH domains was also determined. There were two introns in the bHLH domain in all of the *PIF7* and *PIF8* homologous genes from *B. platyphylla*, *A. thaliana*, and *P. trichocarpa*, and three in the bHLH domain of the other genes except *AtPIF4* (only one intron) and *AtPIF5* (two introns). This result indicated that the *PIFs’* gene structure is very conservative, while the birch *PIF* gene is highly consistent with the poplar *PIF* gene.

We used the Multiple Em for Motif Elicitation (MEME) motif search tool to investigate the motifs shared among related proteins within the same subfamily and identified ten distinct motifs (Figure 2B). Motif 1, the representative bHLH domain, and Motif 3 were identified in all three PIF proteins. Motif 2 and Motif 7 were also identified in all three PIF proteins except PtPIF9a/9b, BpPIF9a/9b. Motifs 1, 3, and 5 were identified in all BpPIF proteins. Motifs 7 and 8 were generally located in the N-terminus of PIF proteins, but motif 9 was in the C-terminus. Interestingly, only *AtPIF2* did not contain Motif 5.

Some of the specific motifs were absent in most PIF proteins. For example, Motif 10 only existed in PtPIF10, AtPIF3, BpPIF3, PtPIF3a, and PtPIF3b. Therefore, these motifs’ functions concerning these proteins need further investigation. In summary, the results of gene structure and conserved motif analyses additionally support the results of phylogenetic analysis, illustrating that the evolution of each subfamily was well conserved in three different species.

### 2.4. Synteny Analysis of the PIF Genes in B. platyphylla., Arabidopsis and P. trichocarpa

**Figure 3:**
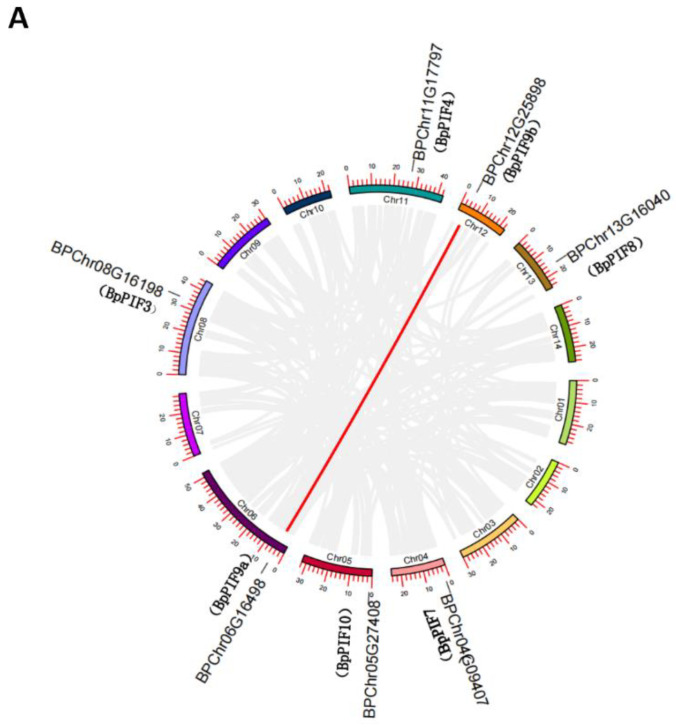

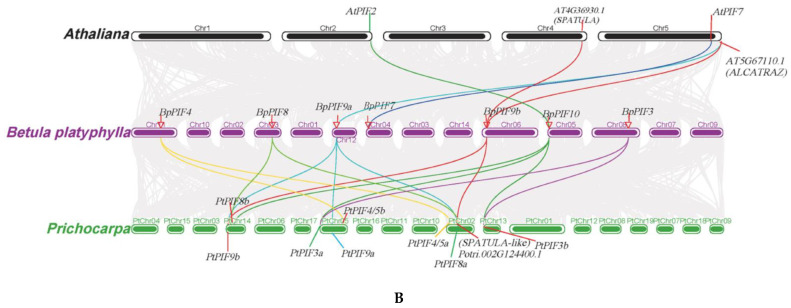
Gene duplication and synteny analysis of *BpPIF* genes. (**A**) Schematic representations for the chromosomal distribution and interchromosomal relationships of *BpPIF* genes. Grey lines indicate all synteny blocks in the *Betula platyphylla* Suk. Genome, and the red lines indicate segmental duplicated *BpPIF* gene pairs. (**B**) Synteny analysis of *BpPIF* genes between *Betula platyphylla*, *Arabidopsis thaliana*, and *Populus trichocarpa*. Grey lines in the background indicate the collinear blocks within Betula, Arabidopsis, and Populus genomes, while the red lines highlight the syntenic *BpPIF* gene pairs. Lines with different colours highlight different gene pairs.

Gene duplication is an important mechanism for acquiring new genes and creating genetic novelty in organisms. Many new gene functions have evolved through gene duplication, which has contributed tremendously to the evolution of developmental programmes in various organisms. Gene duplication can result from unequal crossing over, retroposition, or chromosomal (or genome) duplication [34]. According to the previous results, seven genes were located on the chromosome except for *BpPIF1*, which may be an artefact of the assembly technology. To verify the duplication of these seven *BpPIF* genes, we analysed the syntenic regions using MCscanX software. As shown in Table S5, a total of 2845 tandem duplication gene pairs and 215 segmental duplication blocks (Table S6) were identified in the *Betula platyphylla* genome, respectively. In addition, one segmental duplication event (*BPChr12G25898 and BPChr06G16498*) was also identified in *the B. platyphylla* PIF gene family (Figure 3A, Table S2). This shows that the Asian birch has experienced at least one whole-genome duplication event during evolution.

Our statistical analyses of all 33,966 genes of *B*. *platyphylla* allowed us to divide their origins into five types: dispersed duplication (DD), whole genome (WGD) or segmental duplication (SD), tandem duplication (TD), proximal duplication (PD), and singleton genes. As shown in Figure S1, 52% of the genes may have arisen from transposition (either replicative, non-replicative, or conservative), 16% from WGD or SD, 13% from TD, 10% were singleton, and 9% were from transposition (replicative, non-replicative or conservative).

To further expound on the genomic mechanisms underlying the *BpPIF* family, comparative syntenic maps of *B. platyphylla* associated with Arabidopsis and Populus were constructed (Figure 3B). Four (*BpPIF7*, *BpPIF9a*, *BpPIF9b,* and *BpPIF10*) and six (*BpPIF3*, *BpPIF4*, *BpPIF8*, *BpPIF9a*, *BpPIF9b*, and *BpPIF10*) *BpPIF* genes showed syntenic relationships with those in Arabidopsis and Populus, respectively. Unexpectedly, *BpPIF10* has been associated with *AtPIF2,* a syntenic gene pair from Arabidopsis. *BpPIF9b* has collinearity with SPATULA (AT4G36930.1.) and ALC (AT5G67110.1) genes, which also belong to the bHLH gene family in Arabidopsis. *BpPIF9a* and *BpPIF9b* have collinearity with the ALC gene (AT5G67110.1).

*BpPIF* genes were associated with at least two syntenic gene pairs between *B. platyphylla* and *P. trichocarpa*, indicating that these genes may have played an essential role in the PIF gene family during the evolution of woody plants. However, these orthologous pairs may have already existed before the ancestral divergence. The exception is *BpPIF7,* a gene that is missing in *Populus trichocarpa*. It is speculated that this gene may either be redundant with other genes or have evolved a unique function.

### 2.5. Conserved Motif and Transcription Factor Binding Site Analysis in the Promoter of the BpPIFs

**Figure 4:**
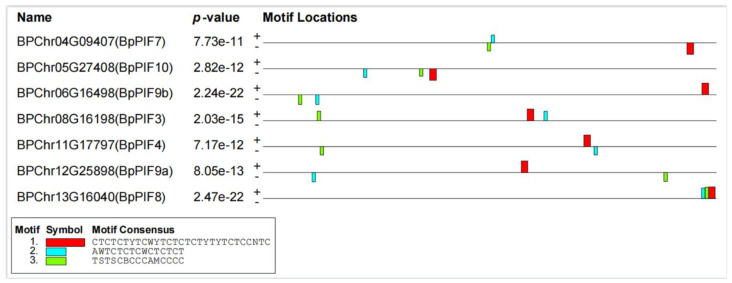
Putative conserved motifs in the promoters of *BpPIFs* according to the phylogenetic relationship. The three motifs were identified online using the MEME with a 2.0 kb upstream region of the start codon of all *BpPIF* genes. The following parameters “nmotifs 3, minw 6, maxw 20, minsites 30, maxsites 100” were used in MEME. Different colours indicate different motifs. The logos of three conserved domain sequences, shown in the top right corner, were obtained from the MEME Suite website.

To analyse conserved sequences potentially involved in regulating *BpPIF* genes, we selected a 2.0 kb upstream region from the start codon of each *BpPIF* gene. The MEME suite identified three conserved motifs in the promoters of all BpPIFs (Figure 4). To know if these motifs were potential TF binding sites, we scanned the promoters of *BpPIFs* using the regulation prediction tool in PlantRegMap. Many TFs possess over-represented targets in the input gene set under cutoff *p*-value ≤ 0.05. Among these TFs, all *BpPIF* genes were candidate targets of BBR/BPC TF (BPChr01G22857 and BPChr03G04964). Interestingly, the positions of BBR/BPC binding sites were consistent with those of three conserved motifs identified by MEME. Furthermore, the three binding motifs were found in promoters of all seven *BpPIF* genes, indicating that BBR/BPC and GRAS TFs may directly bind the one or more conserved motifs in the promoters of BpPIFs to regulate their expression.

**Figure 5:**
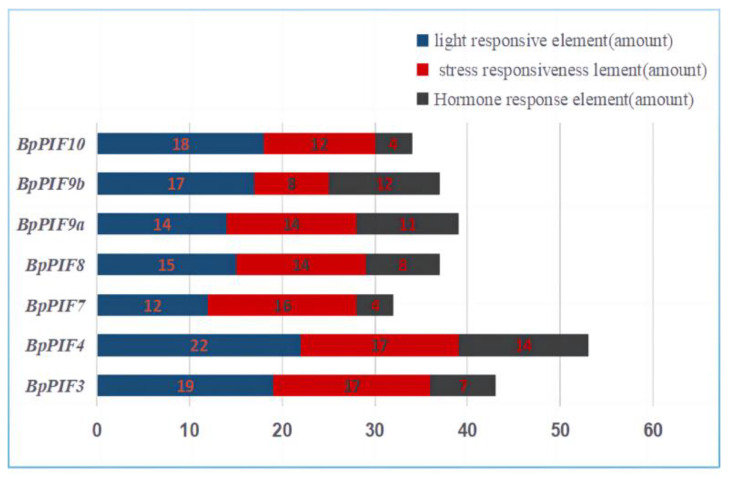
Cis-elements in the promoter of *BpPIF* genes that are related to stress responses and plant development. The X-axis represents the number of cis-acting elements.

Cis-Element analysis in the *PIF* gene promoters and functional prediction of *PIFs* were performed in PlantCARE (Table S7). We counted the number of three types of cis-acting elements: light-responsive, stress-responsive, and hormone-responsive. The predicted cis-elements differed among different genes, but the cis-elements related to photoreaction were the most abundant (3-AF1 binding site, AE-box, AT1-motif, Box 4, G-Box, GATA-motif, GT1-motif, Sp1, TCT-motif, and as-1), which had the largest number in all species (Figure 5). Among them, *BpPIF4* contains the majority of light- and hormone-responsive elements. In addition, we scanned the binding motif of the *BpPIF* promoters and found that there are more than two *BpSPL2* binding motifs GTAC.

### 2.6. Expression Patterns of PIF Genes during Adventitious Root Induction of Transgenic BpSPL2 B. platyphylla and Flower of Naturally Mutated B. platyphylla Based on RNA-Seq

In previous research, we obtained the overexpressed (*35S::BpSPL2*) and suppressed *BpSPL2* (*35S::BpSPL2-SRDX*) transgenic lines. Compared with wild-type (WT) plants, *BpSPL2*-suppressed plants showed root emergence earlier, and the number of ARs and total root length significantly increased (unpublished data). The transcriptomes of wild-type, *35S::BpSPL2*-overexpressed, and *35S::BpSPL2-SRDX*-inhibited expression were sequenced by high-throughput technology at 0, 24, and 96 h after rooting induction with three biological replicates.

**Figure 6:**
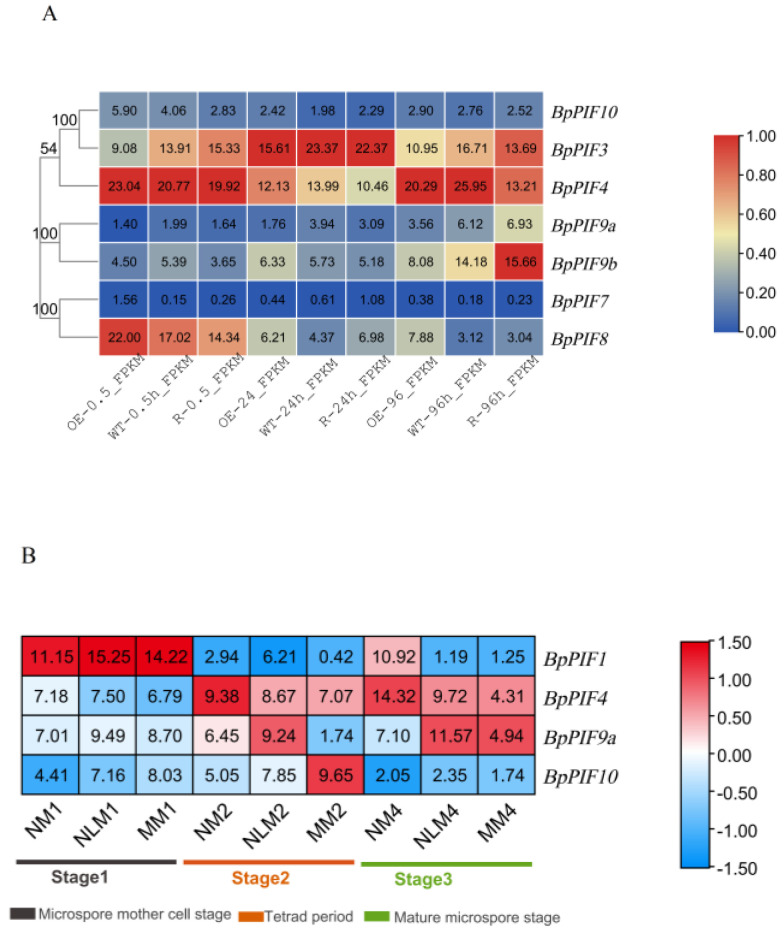
Heatmap of the expression profiles of *BpPIF* family genes (**A**) The developmental expression pattern analysis of *BpPIF* family genes at three developmental stages during rooting induction. The 0.5, 24, and 96 represent 0.5, 24, and 96 h after cutting shoots cultivating in WPM medium; OE-, R-, and WT- represent overexpression lines, suppressed expression lines and wild-type birch, respectively. Clustering was based on log2-transformed FPKM values of seven *BpPIF* genes. (**B**) Expression pattern analysis of four family genes in fertile male flowers and sterile male flowers in different developmental stages of birch. The expression data were acquired from the RNA-seq data with three biological replicates. Values shown on the heatmaps represent the average FPKM value of three biological replicates.

In our RNA-seq, the transcript abundance of one gene (*BpPIF7*) was very low (FPKM < 2.0 in all three stages). The other six *BpPIF* genes (*BpPIF10, BpPIF9b, BpPIF3, BpPIF4, BpPIF9a,* and *BpPIF8*) showed high levels of transcript abundance (FPKM > 2.0) during rooting induction. (Figure 6A). It is worth noting that *BpPIF3* and *BpPIF4* showed the highest expression in all lines at the same time compared to other *BpPIF* genes (Figure 6A).

In addition, we obtained transcriptome data of birch fertile male flowers (NM1, NM2, and NM4) and sterile male flowers (MM1, MM2, and MM4) in different developmental stages. NLM1, NLM2, and NLM4 are semi-sterile inflorescences of different developmental stages on mutant trees. From Figure 6B, we found that in the mature microspore stage, the expression of three *BpPIF* genes (*BpPIF4*, *BpPIF9a,* and *BpPIF10*) was significantly down-regulated in the sterile inflorescence. On the other hand, in the spore mother cell and tetrad stage, the expression level did not change significantly. This result shows that these genes are indispensable in the normal development of male flowers.

### 2.7. The Expression Patterns of Key BpPIF Genes during Root Induction Based on qRT-PCR

**Figure 7:**
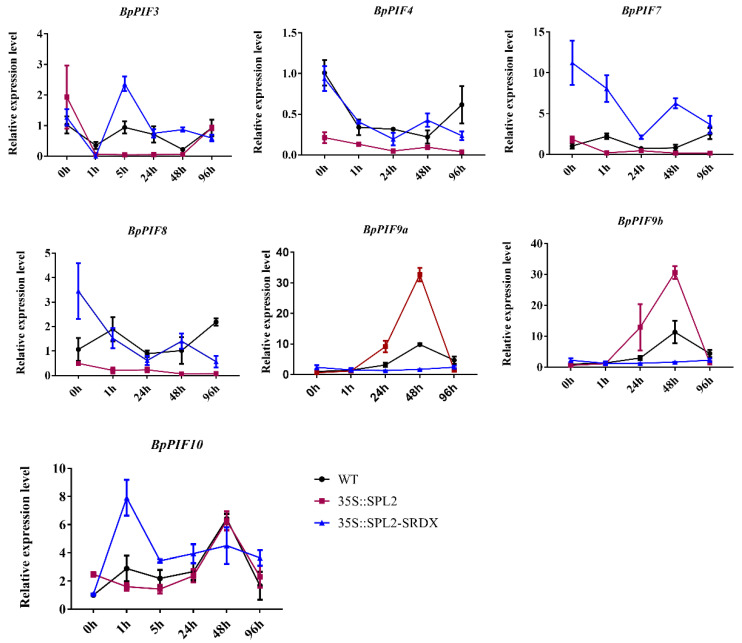
Relative expression levels of *BpPIFs* in adventitious root formation. The number on the X axis indicates the time of rooting induction (hour), The different colours of the lines represent different birch lines. Stars indicate significant differences between OE-, SRDX-, and WT (*p* < 0.05) according to Duncan’s multiple range test, while the equals sign means non-specific difference. Expression levels were calculated based on the 2^−ΔΔCt^ method, with the zero-hour sample chosen as a reference.

To study whether *BpPIFs* correspond to the expression of *BpSPL2* in *B. platyphylla* transgenic lines, we performed RT-qPCR on the *BpPIFs* in the three lines during the adventitious root induction of *B. platyphylla*. From Figure 7, we can see that these genes respond to the expression of *BpSPL2* at different times. The most obvious were *BpPIF3, BpPIF4, BpPIF7, and BpPIF8*. The results showed that *BpPIF3*, *BpPIF4*, *BpPIF7,* and *BpPIF8* had an opposite expression regulation pattern with *BpSPL2* at a certain time of adventitious root occurrence. The phenotype of more adventitious roots in *BpSPL2* inhibited transgenic lines, and less adventitious roots in overexpressed transgenic lines were consistent. This showed that *BpSPL2* strongly inhibited the expression of these genes. The expression level of *BpPIF7* increased in the *BpSPL2*-suppressed-expression lines and decreased in the *BpSPL2*-overexpression lines. It is speculated that *BpPIF7* may be the target gene of *BpSPL2*.

In contrast, the expression of *BpPIF9a* and *BpPIF9b* was up-regulated by *BpSPL2* at 24 h and 48 h after adventitious root induction. In summary, we can confirm *that BpPIF3*, *BpPIF4*, *BpPIF7*, *BpPIF8*, *BpPIF9a,* and *BpPIF9b* play an essential role in the formation of birch adventitious roots. However, it is speculated that *BpSPL2* directly or indirectly regulates the expression of these genes; this requires further experimental verification.

## 3. Discussion

The *PIF* genes belong to a subfamily of the bHLH superfamily. There are 126 bHLH genes in *Arabidopsis*, divided into 26 different subfamilies. The PIF gene family belong to the fifteenth subfamily [49]. The evolutionary analysis suggests that there are only a few bHLH genes from land plants, chlorophytes, and red alga [5,50]. The expansion of modern plant gene families occurred by genome/segment and tandem duplications [51]. With the development of genomics, the *PIF* homologous gene family has been found in many plants. The present work found eight *BpPIF* genes in the *B. platyphylla* genome. Among these *BpPIFs*, *BpPIF9b* has collinearity with SPATULA and ALC genes (Figure 3B), while *BpPIF9a* and *BpPIF9b* have collinearity with ALC. Previous research described that all modern plant bHLH proteins have evolved from these predecessors through many gene duplications. Additionally, *BpPIF9a*, *BpPIF9b*, and *BpPIF10* formed homologous gene pairs with *PIF* genes in Arabidopsis and Populus, indicating that they may have played a pivotal role in evolution.

SPATULA (SPT), a PIF homolog, is one of the first bHLH transcription factors identified to control plant morphogenesis. ALC is also one of the bHLH transcription factors. It is widely expressed and has considerable overlap with SPT in Arabidopsis [52], considered a multifunctional gene [53]. In addition to affecting the development of pistils and fruits, it also regulates the growth of vegetative organs [53]. SPT is also involved in root growth. As homologous genes, SPT and PIF share similar functions to a certain extent. It is speculated that *PIF* genes may also be involved in regulating plant root development. Therefore, later experimental verification is required to ascertain the exact roles of those genes. Notwithstanding, phylogenetic data indicate that they may share a common ancestor.

Studies have shown that the *PIF* genes are mainly involved in photomorphogenesis and thermomorphogenesis in plants. AR formation could be initiated by multiple pathways [54], but the function of PIFs in root development remains unclear. From the data of the *B. platyphylla* transcriptome with overexpressed or suppressed *BpSPL2* (Figure 6), we could infer that the expression of *BpPIF9a/b* positively correlates with *BpSPL2*, while other *BpPIFs* were negatively correlated with its expression. Furthermore, in the promoter analysis, we found that the promoters of three genes (*BpPIF4*, *BpPIF9b*, and *BpPIF10*) have *BpSPL2* binding motifs (GTACAA/GTACGG). Therefore, *BpPIF4*, *BpPIF9b*, and *BpPIF10* may be the candidate target genes involved in the AR formation caused by cutting. Although there have been many achievements in regulating the formation of adventitious roots by protein interaction modules [55,56], the role *BpPIFs* play in forming birch adventitious roots needs further study.

Due to the evolutionary similarity between birch and poplar, using the regulation prediction tool in PlantRegMap, we speculate that BBR/BPC TF (BPChr01G22857 and BPChr03G04964) may bind to the *BpPIF* promoters. However, whether these two transcription factors can directly interact with BpPIFs still needs to be verified.

In the sterile male flowers of *B. platyphylla*, the expression of *BpPIF4*, *BpPIF9a,* and *BpPIF10* in the mature pollen stage of male flower development was down-regulated. Therefore, these genes may be involved in male flower development. However, in *Arabidopsis*, PIF4 is involved in regulating the flowering time, so the specific role these three genes play in birch male sterility is an open question.

In *Arabidopsis*, the PIF gene family is essential in shading response, stress resistance, and flower development, but the *BpPIFs’* function and relationship should be further studied.

## 4. Materials and Methods

### 4.1. Identification of PIF Genes in Betula platyphylla Suk

We downloaded the *Betula platyphylla* genome data at Phytozome (available online: https://phytozome.jgi.doe.gov/pz/portal.html, accessed on 27 August 2021. PIF proteins of *Arabidopsis thaliana* and *Populus trichocarpa* were downloaded from The Arabidopsis Information Resource (TAIR) database (available online: https://www.arabidopsis.org, accessed on 14 July 2021) and Phytozome. Eight *Arabidopsis thaliana* and ten *Populus trichocarpa* PIF proteins were used as query sequences and Blastp searches against the predicted *B. platyphylla* proteins, and the E-value was set to less than 1 × 10^−7^. All candidate genes were further examined by confirming the existence of bHLH domains using the Pfam and Batch CD-Search program. Basic information (PIs, MWs) was predicted through the ExPASy website (https://web.expasy.org/protparam/, accessed on 29 August 2021).

### 4.2. Phylogenetic Analysis

Multiple sequence alignments were performed by Muscle with default parameters. The phylogenetic trees were constructed with the full protein sequences of PIFs using MEGA7.0 (available online: https://www.megasoftware.net/, accessed on 29 August 2021) [57]. The neighbour-joining (NJ) method was used with the following parameters: Poisson correction, pairwise deletion, and bootstrap (1000 replicates; random seed).

### 4.3. Gene Structure Analysis, Conserved Motif Recognition, and Transcription Binding Site Analysis

The DNA and cDNA sequences corresponding to each predicted gene from the Local Database and the gene structures were analysed using the web-based bioinformatics tool GSDS (available online: http://gsds.cbi.pku.edu.cn/, accessed on 30 August 2021) [58]. MEME (Multiple Expectation Maximisation for Motif Elicitation) was used to identify conserved motif structures of BpPIF protein and promoter sequences [59]. PlantCARE webtool (http://bioinformatics.psb.ugent.be/webtools/plantcare/html/, accessed on 30 August 2021) was used to predict the cis-acting elements within 2000 bp upstream of all *BpPIF* genes. 

### 4.4. Chromosomal Distribution and Gene Duplication

Only seven *BpPIF* genes were mapped to *Betula pendula* chromosomes based on physical location information from the database of *Betula pendula* genome using Tbtools (available online: https://github.com/CJChen/TBtools, accessed on 27 August 2021) [60]. The Multiple Collinearity Scan toolkit (MCScanX) was adopted to analyse the gene duplication events with the default parameters [61]. To exhibit the synteny relationship of the orthologous *BpPIF* genes obtained from *Betula pendula*, Arabidopsis, and rice, we constructed syntenic analysis maps using TBtools.

### 4.5. Plant Materials, Treatment, Sample Collection, and RNA-Seq

Four-week-old wild-type (WT), *35S::BpSPL2* (OE), and *35S::BpSPL2-SRDX* (R) tissue culture seedlings of *B. pendula* had stem segments with apical buds cut at the second internode (about 2.5 cm long), without adding hormones. These cuttings were cultivated in WPM solid medium for 0.5 h, 24 h, and 96 h. The sampling site was about 0.4 cm from the base of the stem. Three biological replicates were set for each processing time point, totalling 27 library sequencing samples, 40 seedlings per repetition and three biological replicates for each treatment. Gene expression levels were analysed by employing the fragments per kilobase of exon per million mapped fragments (FPKM) algorithm (unpublished data). Root Transcriptome sequencing was performed by Suzhou GENEWIZ Biotechnology (https://www.genewiz.com.cn/, accessed on 6 November 2021).

All plant material was derived from 5-year-old *B. platyphylla* growing in the birch forest yard of Northeast Forestry University, Heilongjiang, China. Three types of inflorescences, normal male inflorescences (NM), female inflorescences (F), and mutant male inflorescences (MM), were used to establish transcriptomes. MMs are sterile and appear later in development than NMs since microspore development is aborted at the late mononucleate microspore stage [62]. We obtained the transcriptomes of the NM, F, and MM using high-throughput sequencing with a quality assessment of Q20 = 100%. After assembling into contigs with clean reads, we built a unigene library containing the three transcriptomes. The heatmaps were generated using TBtools.

## Figures and Tables

**Table 1:** Putative members of the *BpPIF* gene family of *Betula platyphylla Suk*.

Gene ID (RNA-Seq Data)	Gene ID (Downloaded Data)	Putative PIF Name	Location	ProteinLength/aa	PI	MW (kDa)	Domain
Bpev01.c0000.g0060.mRNA1	---	*BpPIF1*	---	542	5.65	59.38	bHLH_AtPIF_like
Bpev01.c0918.g0013.mRNA1	BPChr08G16198	*BpPIF3*	Chr08:33527696:33532250:−	742	5.87	79.48	bHLH_AtPIF_like
Bpev01.c1708.g0006.mRNA1	BPChr11G17797	*BpPIF4*	Chr11:26928610:26931540:+	487	5.91	53.71	bHLH_AtPIF_like
Bpev01.c0349.g0043.mRNA1	BPChr04G09407	*BpPIF7*	Chr04:1830185:1835072:−	454	9.25	49.70	bHLH_AtPIF_like
Bpev01.c1013.g0001.mRNA1	BPChr13G16040	*BpPIF8*	Chr13:16435535:16440667:−	558	8.36	60.92	bHLH_AtPIF_like
Bpev01.c0015.g0022.mRNA1	BPChr12G25898	*BpPIF9a*	Chr12:3852366:3859065:−	362	5.01	39.40	bHLH_AtPIF_like
Bpev01.c0555.g0004.mRNA1	BPChr06G16498	*BpPIF9b*	Chr06:4115462:4119588:−	440	5.38	48.32	bHLH_AtPIF_like
Bpev01.c1527.g0004.mRNA2	BPChr05G27408	*BpPIF10*	Chr05:1125580:1127552:−	463	8.8	50.94	bHLH_AtPIF_like

## Data Availability

All relevant data are included within this article.

## Supplementary Materials

The following are available online at: https://www.mdpi.com/article/10.3390/ijms232315326/s1.
