# Supplementary figures and images for: Comprehensive Analysis of Betula platyphylla Suk. PIF Gene Family and Their Potential Functions in Growth and Development
Source: Int J Mol Sci. 2022 Dec 5;23(23):15326. doi: 10.3390/ijms232315326 (PMC9738378; doi:10.3390/ijms232315326)

## BETULA PLATYPHYLLA(33966)

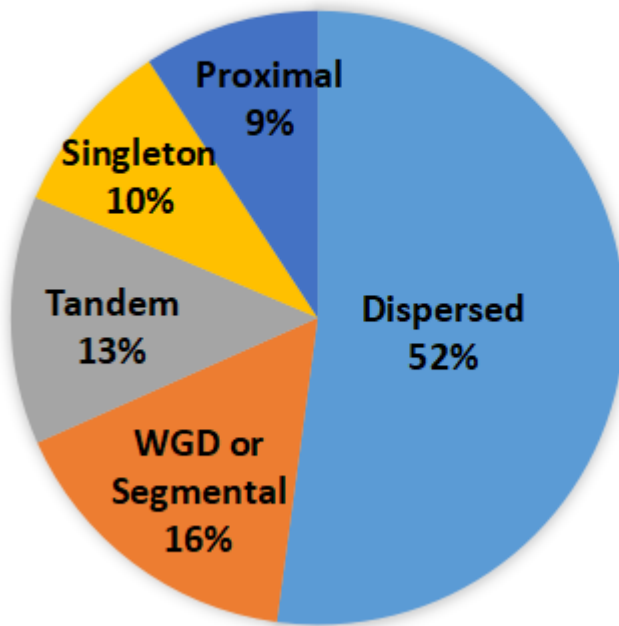

Figure S1: Statistical analyses of all 33,966 genes of *B. platyphylla*

Supplement: Supplementary file 1 [file ijms-23-15326-s001.zip › ijms-1963621-Figue S1.pdf]
